# Authors’ Response

**DOI:** 10.4269/ajtmh.19-0411b

**Published:** 2019-10

**Authors:** Barry A. Finette, Megan McLaughlin, Samuel V. Scarpino, John Canning, Michelle Grunauer, Enrique Teran, Marisol Bahamonde, Edy Quizhpe, Rashed Shah, Eric Swedberg, Kazi Asadur Rahman, Hosenera Khondker, Ituki Chakma, Denis Muhoza, Awa Seck, Assiatta Kabore, Salvator Nibitanga, Barry Heath

**Affiliations:** University of Vermont Robert Larner College of Medicine and University of Vermont Children’s HospitalBurlington, VermontTHINKMD, Inc.Burlington, Vermont; THINKMD, Inc.Burlington, Vermont; Northeastern UniversityBoston, Massachusetts; Physicians Computer CompanyWinooski, Vermont; Universidad San Francisco de QuitoQuito, Ecuador; University of San Francisco de Quito–Ecuador Ministry of Health-AffiliateQuito, Ecuador; Save the Children–USFairfield, Connecticut; Save the Children–International BangladeshDhaka, Bangladesh; UNICEF-Burkina FasoOuagadougou, Burkina Faso; University of Vermont Robert Larner College of Medicine and University of Vermont Children’s HospitalBurlington, VermontTHINKMD, Inc.Burlington, Vermont

Dear Sir,

We thank Ansermino et al.^1^ for their comments on our article, which describes the development and validation of a new integrated mHealth clinical risk assessment and triage recommendation platform (MEDSINC).^2^ Digital mHealth platforms, such as MEDSINC are rapidly expanding in low-, middle-, and high-income health-care markets, most importantly as key “scaling accelerators” for health programs using frontline community health workers to increase health-care access, capacity, and quality of delivery.

Incorporation of evidence-based, data-driven algorithms and WHO-approved guidelines is at the core of the MEDSINC platform. The development of our platform included the review of 395 peer reviewed published articles that focused on clinical assessment protocols/guidelines, digital mHealth platforms, field-based validation studies, as well as analytic and statistical approaches, including inter-rater reliability comparative analysis for the type of field-based validation study we performed. Although the WHO Integrated Management of Childhood Illness guidelines were not specifically “written for machine implementation” they have been applied by many in the development of mHealth platforms through the digitalization of paper-based documents/forms. Our approach merged internationally recognized WHO guidelines with a novel physician-based logic that removed the subjective decisions and assessment dependency made by frontline community health workers during their clinical assessments. Our algorithm development and validation testing approach were approved by United States and in country Institutional Review Board human research committees. In addition, THINKMD also established a Quality Management System following the principles of ISO 13485. The design and development of our clinical algorithms have complied with FDA Software as a Medical Device guideline.^3^ In addition, we have followed (and endorse) the international consortium Principles for Digital Development.^4^

Ansermino et al. raised issues about the transparency of our clinical algorithms. Disclosure limitations of clinical algorithms regularly occur in peer-reviewed medical literature, in particular as it pertains to the development of medical devices, biometric sensors, and now with machine learning (ML) and artificial intelligence (AI) neural network–based algorithms. These devices and ML-AI–based algorithms are developed by research and development teams at both universities and private companies, using validation studies in which sensors, devices, and algorithms are compared with “gold standards” to demonstrate accuracy without disclosing specific algorithms used. We followed and described our “open” method for field-truth validation studies and analysis using physicians as our gold standards, which included a description of the strengths and weaknesses of this approach.

Concern was also expressed by Ansermino et al. about “potential personal bias and commercial interest” influencing the technology development and validation. We agree these are important points to consider. THINKMD is a U.S.-based benefit corporation which by definition is required to develop innovative health technology that has societal impact.^5^ In addition, the legal structure of a U.S.-based benefit corporation does not require its officers to make decisions that maximize profits at the expense of developing validated high-quality technology and promoting impact.^5^ We agree that, unfortunately, there are historical and current examples of biased and monetary priorities influencing health technology, medical device, and pharmaceutical companies. However, these issues should be limited for U.S. benefit corporations. With respect to our work, it is important to note that significant financial support for the validation studies was provided by funds from the Ministries of Health of Ecuador and Burkina Faso, University of San Francisco-Quito medical school, United Nations Children’s Fund (UNICEF), and Save the Children regional/local programs. The central goal of this THINKMD collaboration, which includes senior academic physicians and scientists, international aid agency physicians, and senior public health professionals from UNICEF and Save the Children, was to produce a high-quality validated product and perform transparent validation studies. We believe Ansermino’s concerns regarding “bias and monetary commercial interests” for this research consortium are misplaced.

Ansermino et al., also stressed the importance of “fidelity and rigor of performance” being a priority of WHO/International Telecommunication Union, which we fully support. Indeed, one of our authors (B. A. F.) has participated in two key WHO working groups to address these issues.^6,7^ In addition, the unique design of our clinical logic, and the data that are acquired with its use, allows us to analyze and determine exactly which data points and algorithm logic led to each of the clinical assessments generated. This is an extremely powerful and necessary criterion for determining the quality of the assessments and for continually expanding and improving clinical algorithms with future validation studies.

Ansermino et al. alluded that our clinical algorithms were updated during our validation studies. It is important to restate that all comparative analyses were performed with identical algorithm sets. As we described, we did include new clinical assessment algorithms for eight additional clinical assessments, which we included in our Ecuador and Bangladesh validation studies.

Ansermina et al. also importantly warned about “over-promising” of our technology. We believe we provided an objective presentation and discussion of our work for the medical and scientific community to review and interpret. We enthusiastically look forward to additional discussions, as well as future collaborative partnerships to ensure the rapid development and implementation of accurate and validated digital mHealth technology that will lead to sustainable health impact for those with the greatest need.
